# Longitudinal Blood Pressure Control, Long-Term Mortality, and Predictive Utility of Serum Liver Enzymes and Bilirubin in Hypertensive Patients

**DOI:** 10.1161/HYPERTENSIONAHA.114.04915

**Published:** 2015-06-10

**Authors:** Linsay McCallum, Jeemon Panniyammakal, Claire E. Hastie, Jonathan Hewitt, Rajan Patel, Gregory C. Jones, Scott Muir, Matthew Walters, Naveed Sattar, Anna F. Dominiczak, Sandosh Padmanabhan

**Affiliations:** From the BHF Glasgow Cardiovascular Research Centre, Institute of Cardiovascular and Medical Sciences, University of Glasgow, Glasgow, UK.

**Keywords:** alkaline phosphatase, aspartate transaminase, bilirubin, γ-glutamyl transpeptidase, hypertension, liver function tests, mortality

## Abstract

There is accruing evidence from general population studies that serum bilirubin and liver enzymes affect blood pressure (BP) and cardiovascular risk, but it is unclear whether these have an impact on hypertensive patients in terms of long-term survival or BP control. We analyzed 12 000 treated hypertensive individuals attending a tertiary care clinic followed up for 35 years for association between baseline liver function tests and cause-specific mortality after adjustment for conventional cardiovascular covariates. Generalized estimating equations were used to study the association of liver tests and follow-up BP. The total time at risk was 173 806 person years with median survival 32.3 years. Follow-up systolic BP over 5 years changed by −0.4 (alanine transaminase and bilirubin), +2.1(alkaline phosphatase), +0.9(γ-glutamyl transpeptidase) mm Hg for each standard deviation increase. Serum total bilirubin and alanine transaminase showed a significant negative association with all-cause and cardiovascular mortality, whereas alkaline phosphatase and γ-glutamyl transpeptidase showed a positive association and aspartate transaminase showed a U-shapedassociation. Serum bilirubin showed an incremental improvement of continuous net reclassification improvement by 8% to 26% for 25 year and 35 year cardiovascular mortality, whereas all liver markers together improved continuous net reclassification improvement by 19% to 47% compared with reference model. In hypertensive patients, serum liver enzymes and bilirubin within 4 standard deviations of the mean show independent effects on mortality and BP control. Our findings would support further studies to elucidate the mechanisms by which liver enzymes and bilirubin may exert an effect on BP and cardiovascular risk, but there is little support for using them in risk stratification.

## Abstract 2

Supplemental Digital Content is available in the text.

Liver biochemistry is not only a common screening test in the outpatient and primary care settings, but also increasingly assessed as part of insurance medicals.^1^ Bilirubin has been shown to be an effective antioxidant both in vitro^2^ and in vivo,^3^ positively related to the total serum antioxidant capacity in humans^4^ and suppress the oxidation of LDL cholesterol.^5^ Several population studies have reported a significant negative correlation between serum bilirubin concentrations and blood pressure (BP),^6^ severity of atherosclerosis,^7^ incident coronary heart disease,^8^ and with decreased mortality risk.^9,10^ Interestingly, there is also evidence for both positive and negative relationships with mortality for alanine transaminase (ALT),^11–15^ even though higher, not low, ALT is also considered a marker for nonalcoholic fatty liver disease.^16^ In contrast, γ-glutamyltranspeptidase (GGT) is associated with a positive relationship to mortality and cardiovascular disease.^11,15,17–19^ A potential causal role for bilirubin is supported by studies of human sequence variations in the *UGT1A1* gene, which result in moderate increases in plasma bilirubin and a decreased risk for the development of cardiovascular disease (CVD).^6^ Furthermore, in animal studies, moderate hyperbilirubinemia, induced either by knockout of *UGT1A1* or blockade with indinavir, was associated with a 50% decline in BP response to angiotensin II and no effect on glomerular filtration rate.^20,21^ Several studies in both genetic and experimental models have demonstrated that heme oxygenase-1 induction (which can increase bilirubin levels) can attenuate the development of hypertension, as well as lower BP, in established hypertension.^22,23^ To date, most large studies have analyzed single liver tests, and there is no data on predictive value of these markers for cardiovascular outcomes or the combined effects of different liver tests on outcomes. It is unclear whether the effects of bilirubin and liver enzymes have an impact on hypertensive patients in terms of long-term survival or BP control. In this study, we propose to test whether serum bilirubin and liver enzyme levels within the normal range have independent effects on mortality and BP control and whether any of these tests alone or in combination have incremental predictive value for cardiovascular mortality in treated hypertensive patients.

## Methods

### Study Setting and Study Population

The Glasgow BP clinic provides a secondary and tertiary level service to individuals with hypertension from the West of Scotland. Details of the cohort and clinical measurements and outcome assessment are presented in Methods in the online-only Data Supplement and described previously.^24^

### Statistical Analysis

All analyses were restricted to individuals in the database with liver tests measured for at least one variable (N=12 000) at the registration visit (Figure S1 in the online-only Data Supplement). Analyses were performed in the overall population and in the subset after excluding individuals with high liver test values (values above 4 standard deviations, which are indicative liver injury or disease)—defined as aspartate transaminase (AST) >100 IU/L (n=198, 1.8%), ALT>100 IU/L (n=408, 4.0%), GGT>100 IU/L (n=762, 7.2%), alkaline phosphatase (ALP) >240 IU/L (n=1041, 9.1%), and total bilirubin >30 μmol/L (n=145, 1.3%). The distributions of liver enzymes were reviewed, and all except albumin showed non-normal distributions and were logarithmically transformed for analysis. Pearson correlations between liver enzymes and bilirubin were calculated. Multiple Imputation using Chained Equation was used for imputing missing values of liver test variables (GGT=1546, AST=941, ALT=2168, ALP=638, and total bilirubin =1110) and cholesterol (n=1012). The diagnostics of 10 imputed data sets for each variable were performed and the convergence was tested.

The characteristics of the study population in men and women were compared using independent *t* tests and analysis of variance, where appropriate, for continuous variables and Chi-Square tests for categorical variables. The study population was divided into groups based on quartiles of serum liver enzymes. Cox proportional hazard (Cox-PH) models were used to analyze the influence of baseline liver enzymes on all-cause, CVD, ischemic heart disease, stroke, and non-CVD mortality. The covariates included were sex, baseline age, body mass index, smoking status (never versus ever), systolic BP (SBP), diastolic BP (DBP), total cholesterol, diabetes mellitus status, alcohol use, estimated glomerular filtration rate, and final achieved SBP. A variable on year of first visit strata (epochs) was used to adjust the secular trend in mortality and was divided into 5 categories (first visit 1977 or before, between years 1978 and 1985, 1986 and 1993, 1994 and 2001, and 2002 and thereafter). The proportional hazards assumption was assessed through examination of log-minus-log plots.^25^ Initially, bilirubin and other concomitant liver function tests were assessed as categorical variables in quartiles (Models 1–5) adjusted for each other and the above described covariates. Hazard ratios (HR) were also generated per one standard deviation increase in liver tests and with all other covariates as in the above models. These analyses were repeated after imputing missing data for liver tests and cholesterol. Given the potential nonlinear relationship of the liver function variables and time to mortality, a regression spline model was also set up to further smoothen the hazard functions. The relative log hazard functions with associated 95% confidence intervals (CIs) were plotted in a graph.

Regression modeling with generalized estimating equations was used to study the association of liver biochemistry variables with follow-up BP.^26^ Individuals with at least 4 annual BP assessments in the first 5 years of follow-up and survival up to a minimum of 5 years period were included in this analysis. The association was adjusted for baseline age, sex, alcohol and tobacco use, and estimated glomerular filtration rate. The models were repeated after stratifying the population based on different baseline variables, such as age, body mass index, and alcohol use.

The Cox PH models were fitted to data from all participants and then predictive ability was assessed using measures of risk discrimination and reclassification by fitting models with and without serum bilirubin. Harrel’s c-index, net reclassification improvement (NRI), and Integrated Discrimination Improvement were used to assess discrimination and reclassification (see Methods in the online-only Data Supplement). We computed time-to-event survival–based NRI and category-free reclassification measures (cNRI). The cNRI requires that the subject’s risk probability changes, without any limit, to define an upward or downward reclassification, whereas for NRI, we used risk cut-offs of 0% to 10%, 10% to 20%, and >20%. Integrated Discrimination Improvement is independent of category and considers separately the actual change in calculated risk for each individual for those with and those without events (ie, not merely the direction of change as with the cNRI). Reclassification over different periods of follow-up—10 years, 20 years, and 35 years—were generated. Stata Version 12.0 (Statacorp) was used for all statistical analysis. The R packages nricens and PredictABEL were used for risk discrimination and reclassification analyses.

## Results

### Demographic and Clinical Characteristics of the Study Population

The study population was middle-aged (50.8+14.6), 53% female, overweight (body mass index =27.6+5.8), and hypertensive (SBP=163.9+29.2 and DBP=97.2+19.8). Forty-four percent were smokers and nearly two thirds (60%) drank >6 U of alcohol per week. The BP after 5 years of follow-up (SBP=147.9+22.5 and DBP=87.9+11.9) was significantly lower than the baseline BP. Less than one fifth of the study population first presented to clinic before 1980 (17%) and 22% first presented after 2003. The proportions of Gilbert syndrome (serum bilirubin >17 μmol/L) in men and women were 7.1% and 5.7%, respectively. There were moderate bivariate correlations between AST, ALT, and GGT, but bilirubin and ALP showed little correlations to each other and other enzymes (Figure S2). The full demographic and clinical characteristics are given in Table. Table S1A–S1E provides demographic and clinical characteristics stratified by quartiles of liver enzymes and bilirubin.

**Table.**
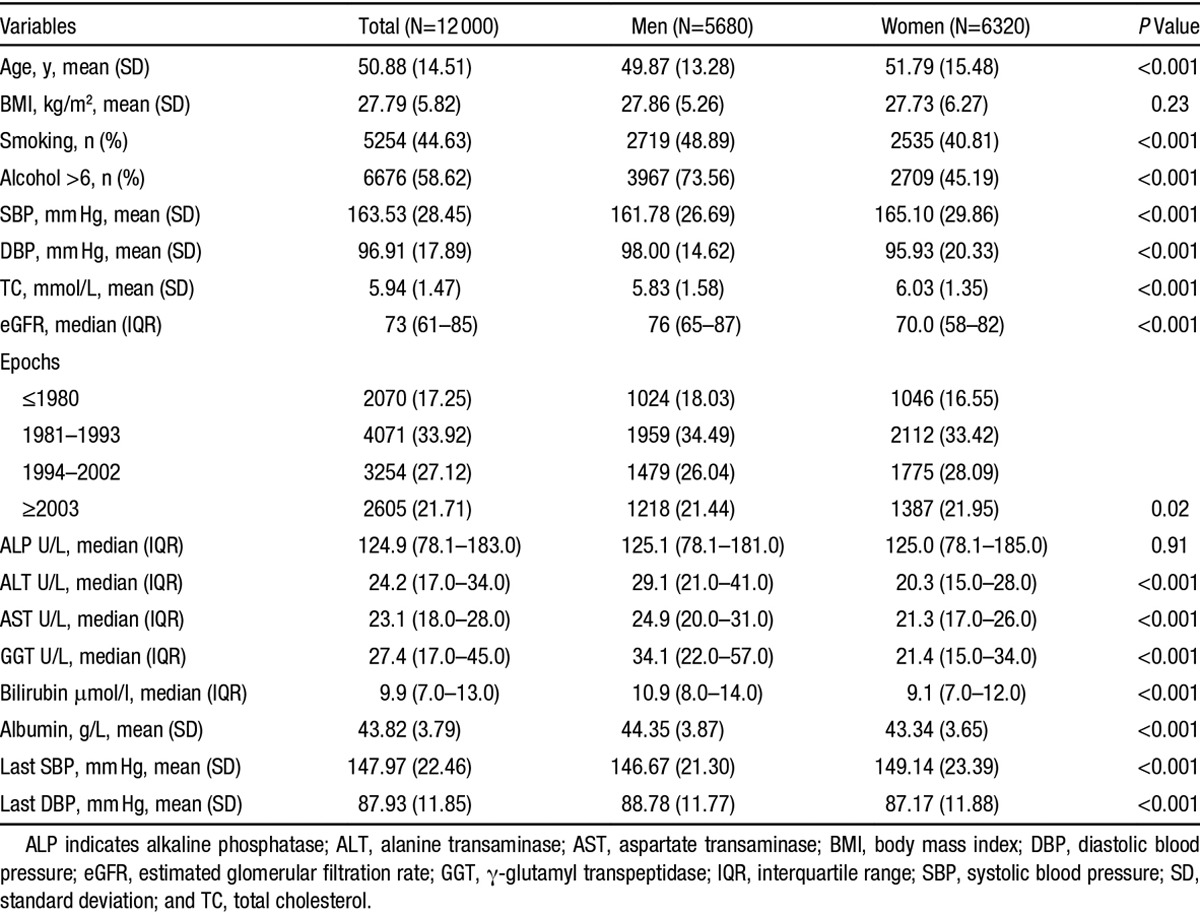
Baseline Characteristics of the Study Population

### Longitudinal Changes in Blood Pressure and Liver Biochemistry

3920 subjects who had at least 3 annual BP recordings in the first 5 years were included in the generalized estimating equations analyses. The median (interquartile range) of the number of annual BP measurements per individual was 5 (4–6). After adjustment for conventional covariates, including ALT, GGT, ALP, and bilirubin, the annual rate of change in BP in the first 5 years of follow-up was −3.1/−1.61 mm Hg (95% CI −3.2 to −2.9/−1.68 to −1.55 mm Hg), and this is considered the treatment effect on BP. The specific effect of ALT and bilirubin on longitudinal BP change was opposite to that of ALP and GGT (Table S2). One SD increase in ALT and bilirubin was associated with a 0.4 mm Hg decrease in SBP over 5 years after accounting for the annual reduction in BP because of treatment effects and the effect of other covariates (Figure S3). In contrast, each SD increase in ALP or GGT was associated, respectively, with a 2.1 or 0.9 mm Hg rise in SBP over 5 years. The BP effects of bilirubin and ALT were clearly apparent in subgroups who were >55 years old or body mass index >25 or consumed >6 U/week of alcohol, whereas the BP effect of ALP and GGT were consistent in all subgroups. An effect on DBP was observed only for ALP and GGT.

### Survival Analysis and Risk Prediction

The total time at risk was 173 806 person years (p-y) with median survival time of 32.2 years from initial clinic appointment. The incidence rates were 17.59 (95% CI, 16.98–18.22), 10.08 (95% CI, 9.62–10.56), 5.40 (95% CI, 5.07–5.76), 2.50 (95% CI, 2.27–2.74), and 7.51 (95% CI, 7.11–7.93) per 1000 p-y of follow-up for all-cause, CVD, ischemic heart disease, stroke, and non-CVD mortality outcomes, respectively.

In the multivariable Cox-PH model, each SD increase in ALT and bilirubin were associated with 14% (HR=0.86; 95% CI, 0.81–0.95) and 11% (HR=0.89; 95% CI, 0.85–0.94) decrease in all-cause mortality, respectively. Similarly, each SD increase in GGT and ALP were associated with 11% (HR=1.11; 95% CI, 1.04–1.18) and 25% (HR=1.25; 95% CI, 1.18–1.33) increase in all-cause mortality, respectively. The imputation of missing variables did not affect the results (Figure S4). Bilirubin and ALT quartiles in the multivariable adjusted Cox-PH model showed a clear negative relationship with all-cause, CVD, and non-CVD mortality (Table S3). The cubic spline regression plots for all-cause mortality are presented in Figure.

**Figure.**
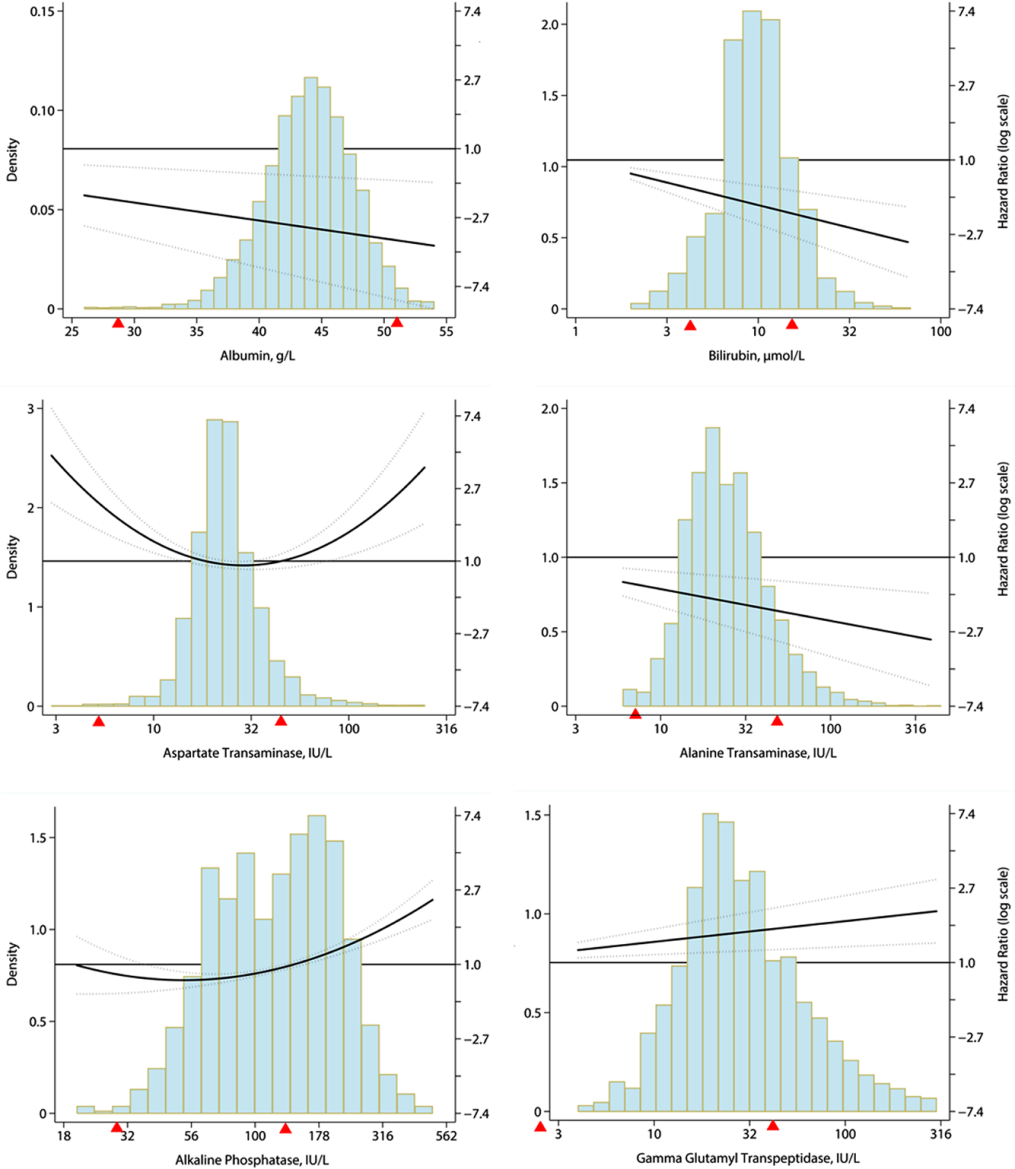
Regression spline Cox proportional hazard model for liver biochemistry parameters and all-cause mortality. The histogram describes the distribution of liver parameters. The hazard ratios are given in the secondary *Y* axis. The thick bold line represents the point estimate (hazard ratio) for the variables on *X* axis (liver parameters) and the light grey lines represent the 95% confidence intervals.

The sequential addition of different liver biochemistry measurements singly or in combination produced trivial or no change in the C statistic (Table S5). The addition of a single liver biochemistry to the risk models showed bilirubin improved cNRI by 16.5% and 17.5%, respectively, for 35 year and 20 year CV mortality, but not for 10 year mortality. Inclusion of bilirubin and ALP resulted in a 29.9%, 30.4%, and 21.9% improvement in cNRI for 35 year, 20 year, and 10 year CV mortality, respectively, and this was similar when all the liver biochemistry tests were included in the model. The corresponding Integrated Discrimination Improvements were 1.5%, 1.1%, and 0.88% for 35 year, 20 year, and 10 year CV mortality, respectively. Full risk discrimination and reclassification analyses are presented in Table S6.

## Discussion

In the largest single cohort study of all liver biochemistry parameters (AST, ALT, GGT, ALP, bilirubin, and albumin) within the normal range, we show in treated hypertensive patients that the tests comprising the standard liver biochemistry panel have characteristic-independent effects on mortality, follow-up BP and a modest improvement in cardiovascular mortality prediction. Our findings are complementary to a recent large meta-analysis of liver enzymes mainly in the general population (GGT, ALP, ALT, and AST) from Kunutsor et al,^15^ who showed a log-linear relationship between GGT and ALP with CVD risk and variable association between ALT and CVD end points. We show a negative association with mortality and longitudinal BP for serum bilirubin and ALT, but a positive association for GGT and ALP. Indeed higher baseline ALP and GGT showed a consistent relationship with higher follow-up SBP. Serum bilirubin and other liver function markers offered modest predictive utility (primarily for continuous NRI) over standard clinical risk factors in hypertensive patients.

Bilirubin and ALT show similar associations with mortality and longitudinal BP, but the mechanisms are likely to be different. Bilirubin levels are strongly determined by genetic and environmental influences.^6,27^ The protective effect of bilirubin is supported by data that *UGT1A1* genetic variants underlying Gilbert’s syndrome are also protective against CVD.^8^ The antioxidant and anti-inflammatory properties of bilirubin may partially explain the biological mechanisms associated with this negative relationship.^5^

In our study, higher bilirubin is associated with a lower baseline BP and greater longitudinal reduction in BP, which suggest that the protective effect of bilirubin can be partly explained by BP effects. For example, each μmol/L increase in serum bilirubin decreased the SBP by 0.13 mm Hg. There is considerable physiological evidence showing increases in bilirubin affects renal hemodynamics and thus can decrease BP. For example, increases in bilirubin protect against excessive tubuloglomerular feedback–mediated afferent arteriole vasoconstriction and can attenuate constriction to high levels of vasoconstrictors, such as Ang II.^28^ Moreover, bilirubin is one of the most potent antioxidants in the body,^29^ and Ang II–mediated superoxide production is significantly attenuated in aortic ring segments from moderately hyperbilirubinemic mice.^21^ The reduction of superoxide production in the vasculature by bilirubin is associated with the increase in the bioavailability of nitric oxide as reflected in the nitrate/nitrite levels in the plasma of chronically infused Ang II hypertensive mice made moderately hyperbilirubinemic.^21^

Our finding of a negative relationship between ALT and mortality is independent of all other concomitant liver tests. Previous studies, including a large systematic review, have shown variable associations.^12,13,15,30–32^ The reason for the increased risk seen with lower ALT is not clear. A recent study in the elderly (>70 years) showed that the increased risk associated with low ALT disappeared after adjustment for frailty,^33^ indicating that ALT may be a marker of aging and frailty. ALT levels are known to reduce with increasing age, suggesting that ALT may be a marker of functioning hepatocyte (and muscle mass) because ALT is primarily produced by the liver (with a small proportion from skeletal muscle). If this is true, then we speculate that the observed greater reduction in BP in this cohort with higher ALT levels may indicate better antihypertensive efficacy because the commonly used antihypertensive drugs, ACEI and ARBs, are prodrugs which require hepatic activation. Despite strong correlation between AST and ALT,^34^ the pattern of risk is U-shaped with AST in contrast to ALT. However, we are limited by our data to dissect this further.

Both ALP and GGT are associated with increased mortality risk, which is significant only at the higher end of the distribution. The novel finding in our study is the consistent association of both GGT and ALP with higher longitudinal BP. The magnitude of BP increase is higher with ALP than GGT, and this increase is over and above the 4 mm Hg annual decline in SBP seen in this cohort, which is related to antihypertensive treatment. GGT has pro-oxidant effects because of its role in the extracellular catabolism of glutathione,^35^ and high GGT has been associated with incident diabetes mellitus^36^ metabolic syndrome and fatty liver.^37^ Recent prospective studies and a systematic review show that high GGT is positively associated with increased mortality or incidence of CVD,^11,15,17–19^ and our results reflect this. However, we do not see a clear association between GGT and cardiovascular mortality once the effects of other liver tests are accounted for in the model. There is a linear increase of bilirubin with GGT levels, and the 24% excess risk of cardiovascular mortality associated with the highest quartile of GGT disappears once bilirubin is included in the model. The pro-oxidant and antioxidant properties, respectively,of GGT and bilirubin associated with opposite effects on cardiovascular risk when considered jointly indicates that GGT does not have a major independent impact on cardiovascular mortality. This is also supported by the lack of any incremental predictive utility when GGT is added to conventional clinical risk factors.

The effect of bilirubin and ALT on longitudinal SBP seems to be more prominent in the older age, in the overweight, and in those who consume alcohol, whereas both ALP and GGT show consistent effect on BP change in all subgroups in the opposite direction. It is possible that the variation in SBP seen with ALT and bilirubin may be because of alteration in pharmacokinetic or pharmacodynamic parameters that are reflected in ALT and bilirubin levels. The results for DBP indicate that both ALT and bilirubin have no effect, whereas there is a clear positive effect of ALP and GGT. There are 2 explanations for these findings: (1) the annual reduction in DBP is much lower than the annual reduction in SBP, so we may not be powered to find any additional BP lowering effect of bilirubin or ALT; (2) bilrubin and ALT do not have a truly causal effect on DBP change, and their effects on outcomes are unrelated to their effect on BP. Nevertheless, these are interesting findings showing the heterogeneity in liver biochemistry effects on BP and require validation in independent controlled studies.

The strengths of the current study include a large cohort of nearly 12 000 hypertensive adults, 35 years of follow-up, high event rates, inclusion of the entire circulating range of liver enzymes (except levels suggestive of significant hepatocyte damage or obstructive liver disease). The generalizability of our findings is limited to middle-aged hypertensive patients of European ancestry. We acknowledge the exclusion of individuals without liver enzymes assessed at baseline from our analysis. However, other baseline demographic characteristics of the excluded population were not different from those included in the study (data not shown). The long period over which our cohort was recruited means there will be variation in laboratory methods and reference values over time. We incorporated a variable on year of first visit strata (epochs) to adjust for secular trends in mortality and biochemical profiles. We have used a nonconventional metric of alcohol use because the coding for alcohol use was not consistent over time—this precluded analyses of alcohol dose effect. One of the inherent limitation of the continuous NRI is that it captures minor shifts in probabilities of outcomes and may be challenging to interpret in terms of clinical utility.^38^ Finally, most of the patients were on combination antihypertensive therapy, and the BP control reflects overall BP reduction in a tertiary care clinic and does not inform on the effect of specific antihypertensive drug class.

In conclusion, we confirm independent effects on mortality for liver enzymes and bilirubin within a range of 4 standard deviations from the mean in treated hypertensive patients. This merits further studies to elucidate the mechanisms underlying these results and explore the utility of these inexpensive markers for risk stratification and predicting antihypertensive treatment response.

### Perspectives

The study confirms that serum bilirubin and ALT are negatively associated, whereas ALP and GGT are positively associated with mortality in treated hypertensive patients. Moreover, we show that higher serum ALP and GGT show consistently higher follow-up SBP. The implications for long-term BP control in outpatient clinical practice where these routinely performed inexpensive tests include stratifying hypertensive patients for more intensive follow-up or treatment escalation. Our findings warrant further studies to elucidate their underpinning mechanisms. We also show that in treated hypertensive patients, the predictive potential for cardiovascular mortality with bilirubin and liver enzymes over conventional risk factors may be limited.

## Acknowledgments

We thank the patients and staff at the Glasgow Blood Pressure Clinic at the Western Infirmary in Glasgow and National Health Service Greater Glasgow and Clyde.

## Sources of Funding

This work is supported by a BHF Clinical Research Training Fellowship FS/14/52/30901 (L. McCallum, S. Padmanabhan) and a Wellcome Trust career development fellowship award (J. Panniyammakal) through PHFI-UKC (Public Health Foundation of India and United Kingdom Universities Consortium).

## Disclosures

None.

## Supplementary Material

## Novelty and Significance

### What Is New?

- The study shows that in treated hypertensive patients, serum bilirubin and alanine transaminase are negatively associated, whereas alkaline phosphatase and γ-glutamyltranspeptidase are positively associated with mortality, and aspartate transaminase show a U-shaped relationsip. Higher serum bilirubin and alanine transaminase are associated with lower follow-up blood pressure in contrast to alkaline phosphatase and γ-glutamyltranspeptidase.

### What Is Relevant?

- Baseline bilirubin and liver enzymes show independent effects on mortality for liver enzymes and bilirubin within a range of 4 standard deviations from the mean in treated hypertensive patients. Higher baseline alkaline phosphatase and γ-glutamyltranspeptidase show a positive association with higher follow-up blood pressure predicting poor blood pressure control.

### Summary

This study confirms the previously described differential association of various liver biochemistry variables on outcomes in treated hypertensive patients and additionally demonstrates a similar association with longitudinal blood pressure. The predictive utility of liver enzymes and bilirubin over and above conventional Framingham risk factors is limited,+ and thus, there is little support for using liver enzymes and bilirubin in any risk stratification strategies.
