# Supplementary material for: Longitudinal Blood Pressure Control, Long-Term Mortality, and Predictive Utility of Serum Liver Enzymes and Bilirubin in Hypertensive Patients
Source: Hypertension. 2015 Jun 10;66(1):37–43. doi: 10.1161/HYPERTENSIONAHA.114.04915 (PMC4461392; doi:10.1161/HYPERTENSIONAHA.114.04915)
Supplement: Supplementary file 1 [file hyp-66-37-s001.docx]

**Supplementary Material**

**Longitudinal Blood Pressure Control, Long-Term Mortality and Predictive Utility of Serum Liver Enzymes and Bilirubin in Hypertensive Patients**

Linsay McCallum,* Jeemon Panniyammakal,* Claire E Hastie, Jonathan Hewitt, Rajan Patel, Gregory C Jones, Scott Muir, Matthew Walters, Naveed Sattar, Anna F Dominiczak, Sandosh Padmanabhan

BHF Glasgow Cardiovascular Research Centre

Institute of Cardiovascular and Medical Sciences,

University of Glasgow,

Glasgow, G12 8TA, UK

**Short title:** Liver markers and outcomes in hypertension

^*^ Equal contribution

**Corresponding author:**

Sandosh Padmanabhan,

Institute of Cardiovascular and Medical Sciences,

University of Glasgow,

Glasgow, G12 8TA, UK

Email: [sandosh.padmanabhan@glasgow.ac.uk](mailto:sandosh.padmanabhan@glasgow.ac.uk)

Telephone: +44 141 330 2228

Fax: +44 141 330 6997

**Supplementary Methods**

**The Glasgow blood pressure clinic**

The Glasgow blood pressure clinic (GBPC) provides a secondary and tertiary level service to individuals with hypertension from the West of Scotland. Data from patients attending the clinic are stored in a computerised computerized database, which contains information on individuals attending the clinic from the mid 1970s until 2011. All patients are treated at GBPC until they achieve target BP and are maintained at that level for at least 3 months. Currently the database has information on >16,000 hypertensive patients. The West of Scotland research ethics service (WoSRES) of the National Health Service approved the study of the GBPC database (11/WS/0083).

**Clinical measurements**

Blood pressure measurements were taken manually 3 times, using standardized sphygmomanometers at each visit by specialist hypertension nurses; the mean of the last 2 measurements was recorded at each visit. Patients attending the clinic were advised to take their regular medications as usual. Height and weight of all patients were measured using standardized equipment during each visit. Blood samples were collected at baseline and at regular intervals for estimation of routine hematological and biochemical indices, including liver function and renal function tests. All biochemical investigations were performed, at the Western Infirmary clinical laboratory service, on blood samples obtained at the first visit as part of routine screening. While the liver biochemistry were performed over a long-time scale, the assays were performed in the certified hospital laboratories on automated analyzers with robust attention to external quality control schemes (NEQAS [National External Quality Assessment Service]). Estimated glomerular filtration rate (eGFR) was calculated from baseline serum creatinine values using the Modification of Diet in Renal Disease Study Group (MDRD) equation.^1^ A structured format was used to measure tobacco (any versus none) and alcohol use (quantity and frequency of consumption). All data were electronically captured and maintained as a large single database.

**Outcome assessment**

Records kept by the General Register Office for Scotland ensured notification of a subject’s death (provided that it occurred in the United Kingdom) together with the primary cause of death according to the International Classification of Diseases, 10th Revision, Version for 2007 (ICD-10), codes. We considered cardiovascular deaths (CVD mortality; ICD-10 codes I00-I99), ischemic heart disease deaths (IHD mortality; ICD-10 codes 120-I25), and stroke deaths (stroke mortality; ICD-10 codes I60-I69) in the analysis. Deaths coded out with the aforementioned codes are classified as non-CVD deaths. Mortality data were collected up to April 2011, allowing a maximum of 35 years for participants who had been under follow up for the longest time.

**NRI and IDI**

The NRI value indicates the percentage improvement in correctly re-classifying the outcome (mortality) after incorporation of the new variable in the model. NRI is interpreted as the proportion of patients reclassified to a more appropriate risk category. cNRI counts the direction of change for every individual rather than the crossing of a threshold. Each patient is counted as either +1 or −1 depending on whether the change in calculated risk was in the correct direction (higher for those with events, lower for those without events).

Reference List

(1) Levey AS, Bosch JP, Lewis JB, Greene T, Rogers N, Roth D. A more accurate method to estimate glomerular filtration rate from serum creatinine: a new prediction equation. Modification of Diet in Renal Disease Study Group. *Ann Intern Med*. 1999;130:461-470.

Table S1A: Baseline characteristics of the study population, stratified by ALT quartile

| **Variables** | **ALT<=16 U/L**  **(n=2168)** | **ALT 17-24 U/L**  **(n=3034)** | **ALT 25-34 U/L**  **(n=2184)** | **ALT>=35 U/L**  **(n=2331)** | ***p* value** |
| --- | --- | --- | --- | --- | --- |
| Age yrs, mean (SD) | 53.24 (16.38) | 52.80 (14.45) | 50.83 (12.50) | 47.89 (12.11) | <0.001 |
| Men, n(%) | 578 (26.7) | 1212 (40.0) | 1232 (56.4) | 1618 (69.4) | <0.001 |
| BMI kg/m2, mean (SD) | 26.21 (5.44) | 27.41 (6.09) | 28.25 (5.48) | 29.22 (5.57) | <0.001 |
| Smoking, n(%) | 950 (45.1) | 1316 (44.0) | 962 (44.7) | 1022 (44.9) | 0.881 |
| Alcohol >6 units, n(%) | 1064 (51.9) | 1582 (54.6) | 1325 (63.7) | 1591 (71.8) | <0.001 |
| SBP mmHg, mean (SD) | 167.45 (29.72) | 166.60 (28.68) | 164.20 (27.77) | 161.08 (25.93) | <0.001 |
| DBP mmHGg, mean (SD) | 96.07 (15.29) | 96.73 (14.92) | 98.19 (23.23) | 98.36 (13.94) | <0.001 |
| TC mmol/l, mean (SD) | 5.84 (1.33) | 5.92 (1.27) | 6.01 (1.92) | 6.08 (1.51) | 0.011 |
| eGFR<60, n(%) | 667 (31.0) | 834 (27.7) | 444 (20.5) | 355 (15.4) | <0.001 |
| ALP U/L, median(IQR) | 113 (71-174) | 123 (78-182) | 122 (78-180) | 121 (78-184) | <0.001 |
| AST U/L, median(IQR) | 18 (16-22) | 21 (18-25) | 25 (21-29) | 31 (26-40) | <0.001 |
| GGT U/L, median(IQR) | 18 (13-26) | 23 (16-35) | 31 (21-48) | 46 (28-80) | <0.001 |
| Bilirubin µmol/l, median (IQR) | 9 (7-11) | 9 (7-12) | 10 (8-13) | 11 (8-14) | <0.001 |
| Albumin g/L, mean (SD) | 42.81 (3.66) | 43.40 (3.95) | 44.10 (3.51) | 44.61 (3.64) | <0.001 |

SD=standard deviation, BMI=body mass index, SBP=systolic blood pressure, DBP=diastolic blood pressure, TC=total cholesterol, eGFR=estimated glomerular filtration rate, ALT=alanine aminotransferase.

Table S1B: Baseline characteristics of the study population, stratified by AST quartile

| **Variables** | **AST<=18 U/L (n=2794)** | **AST 19-22 U/L**  **(n=2582)** | **ALT 23-28 U/L**  **(n=2945)** | **AST>=29 U/L**  **(n=2738)** | ***p* value** |
| --- | --- | --- | --- | --- | --- |
| Age yrs, mean (SD) | 49.99 (14.98) | 52.41 (15.07) | 52.11 (14.16) | 49.69 (13.25) | <0.001 |
| Men, n(%) | 916 (32.8) | 1084 (42.0) | 1535 (52.1) | 1715 (62.3) | <0.001 |
| BMI kg/m2, mean (SD) | 27.39 (6.07) | 27.38 (5.54) | 27.87 (5.54) | 28.49 (5.79) | <0.001 |
| Smoking, n(%) | 1277 (46.5) | 1152 (45.4) | 1250 (43.5) | 1188 (44.3) | 0.105 |
| Alcohol >6 units, n(%) | 1458 (54.8) | 1351 (55.1) | 1609 (58.0) | 1769 (68.5) | <0.001 |
| SBP mmHg, mean (SD) | 165.28 (29.68) | 164.29 (28.18) | 164.03 (27.47) | 163.07 (27.60) | 0.406 |
| DBP mmHGg, mean (SD) | 96.97 (15.37) | 96.03 (14.15) | 96.86 (14.39) | 98.57 (21.62) | 0.112 |
| TC mmol/l, mean (SD) | 5.87 (1.31) | 5.90 (1.26) | 5.91 (1.28) | 6.06 (1.80) | 0.325 |
| eGFR<60, n(%) | 704 (25.5) | 670 (26.2) | 723 (24.8) | 515 (19.0) | <0.001 |
| ALP U/L, median(IQR) | 114 (71-173) | 126 (79-180) | 131 (82-187) | 135 (83-193) | <0.001 |
| ALT U/L, median(IQR) | 16 (13-21) | 20 (16-25) | 26 (20-34) | 40 (28-55) | <0.001 |
| GGT U/L, median(IQR) | 20 (14-30) | 23 (16-35) | 27 (18-42) | 45 (26-79) | <0.001 |
| Bilirubin µmol/l, median (IQR) | 9 (6-11) | 10 (7-12) | 10 (8-13) | 11 (8-14) | <0.001 |
| Albumin g/L, mean (SD) | 43.13 (3.84) | 43.60 (3.40) | 44.09 (3.67) | 44.59 (3.65) | <0.001 |

SD=standard deviation, BMI=body mass index, SBP=systolic blood pressure, DBP=diastolic blood pressure, TC=total cholesterol, eGFR=estimated glomerular filtration rate, AST=aspartate transaminase.

Table S1C: Baseline characteristics of the study population, stratified by GGT quartile

| **Variables** | **GGT<=18 U/L (n=2385)** | **GGT 19-22 U/L**  **(n=3040)** | **ALT 23-28 U/L**  **(n=2384)** | **GGT>=29 U/L**  **(n=1980)** | ***p* value** |
| --- | --- | --- | --- | --- | --- |
| Age yrs, mean (SD) | 48.75 (16.68) | 52.51 (15.03) | 52.42 (13.80) | 51.07 (12.78) | <0.001 |
| Men, n(%) | 562 (23.6) | 1266 (41.6) | 1366 (57.3) | 1273 (64.3) | <0.001 |
| BMI kg/m2, mean (SD) | 26.25 (5.32) | 27.82 (5.84) | 28.50 (5.93) | 29.44 (6.09) | <0.001 |
| Smoking, n(%) | 830 (35.2) | 1249 (41.8) | 1138 (48.5) | 997 (51.6) | <0.001 |
| Alcohol >6 units, n(%) | 1064 (46.8) | 1468 (51.0) | 1313 (58.3) | 1258 (67.8) | <0.001 |
| SBP mmHg, mean (SD) | 159.94 (28.31) | 162.31 (27.82) | 162.72 (27.18) | 161.94 (26.53) | 0.164 |
| DBP mmHGg, mean (SD) | 94.59 (14.37) | 95.12 (19.23) | 96.05 (13.58) | 97.79 (23.69) | <0.001 |
| TC mmol/l, mean (SD) | 5.68 (1.25) | 5.83 (1.26) | 5.90 (1.50) | 6.14 (1.28) | <0.001 |
| eGFR<60, n(%) | 565 (24.1) | 777 (26.0) | 545 (23.2) | 382 (19.6) | <0.001 |
| ALP U/L, median(IQR) | 108 (70-159) | 133 (84-184) | 141 (87-194) | 146 (92-200) | <0.001 |
| ALT U/L, median(IQR) | 18 (14-23) | 21 (16-28) | 26 (19-35) | 32 (23-46) | <0.001 |
| AST U/L, median(IQR) | 20 (17-24) | 21 (18-26) | 23 (19-28) | 27 (21-34) | <0.001 |
| Bilirubin µmol/l, median (IQR) | 9 (7-12) | 10 (7-13) | 10 (8-13) | 10 (8-13) | <0.001 |
| Albumin g/L, mean (SD) | 43.36 (3.86) | 43.65 (3.65) | 44.17 (3.48) | 44.32 (3.62) | <0.001 |

SD=standard deviation, BMI=body mass index, SBP=systolic blood pressure, DBP=diastolic blood pressure, TC=total cholesterol, eGFR=estimated glomerular filtration rate, GGT=gamma-glutamyltransferase.

Table S1D: Baseline characteristics of the study population, stratified by ALP quartile

| **Variables** | **ALP<=78 U/L (n=2751)** | **ALP 79-125 U/L (n=2957)** | **ALP 126-183 U/L (n=2828)** | **ALP>=184 U/L**  **(n=1849)** | ***p* value** |
| --- | --- | --- | --- | --- | --- |
| Age yrs, mean (SD) | 48.31 (13.88) | 50.67 (13.86) | 51.14 (14.52) | 52.94 (14.87) | <0.001 |
| Men, n(%) | 1302 (47.3) | 1409 (47.7) | 1378 (48.7) | 925 (50.0) | 0.266 |
| BMI kg/m2, mean (SD) | 26.85 (5.20) | 27.43 (5.59) | 28.18 (5.68) | 28.64 (6.36) | <0.001 |
| Smoking, n(%) | 1075 (40.3) | 1310 (45.3) | 1194 (42.8) | 880 (48.1) | <0.001 |
| Alcohol >6 units, n(%) | 1801 (69.1) | 1788 (63.3) | 1449 (54.7) | 935 (53.5) | <0.001 |
| SBP mmHg, mean (SD) | 164.66 (28.23) | 165.93 (29.17) | 160.33 (26.96) | 163.70 (27.65) | <0.001 |
| DBP mmHGg, mean (SD) | 98.04 (15.40) | 98.01 (14.72) | 96.8 (20.88) | 96.31 (14.11) | <0.001 |
| TC mmol/l, mean (SD) | 6.00 (1.50) | 6.03 (1.31) | 5.80 (1.21) | 5.91 (1.99) | 0.002 |
| eGFR<60, n(%) | 565 (24.1) | 777 (26.0) | 545 (23.2) | 382 (19.6) | <0.001 |
| ALT U/L, median(IQR) | 22 (16-34) | 24 (17-35) | 23 (17-34) | 24 (18-35) | <0.001 |
| AST U/L, median(IQR) | 22 (17-28) | 23 (18-28) | 23 (19-29) | 24 (20-30) | <0.001 |
| GGT U/L, median(IQR) | 22 (14-36) | 26 (17-43) | 27 (18-46) | 30 (20-49) | <0.001 |
| Bilirubin µmol/l, median (IQR) | 9 (7-12) | 9 (7-12) | 11 (8-14) | 10 (8-13) | <0.001 |
| Albumin g/L, mean (SD) | 43.46 (4.05) | 43.56 (3.76) | 44.28 (3.39) | 44.29 (3.59) | <0.001 |

SD=standard deviation, BMI=body mass index, SBP=systolic blood pressure, DBP=diastolic blood pressure, TC=total cholesterol, eGFR=estimated glomerular filtration rate, ALP=alkaline phosphatase.

Table S1E: Baseline characteristics of the study population, stratified by bilirubin quartile

| **Variables** | **Bili<=7** µmol/l  **(n=2845)** | **Bili 8-10** µmol/l  **(n=3348)** | **Bili 10.1-13** µmol/l  **(n=2381)** | **Bili>=13.1** µmol/l  **(n=2316)** | ***p* value** |
| --- | --- | --- | --- | --- | --- |
| Age yrs, mean (SD) | 50.88 (14.60) | 51.70 (14.56) | 51.37 (14.23) | 50.10 (14.50) | 0.587 |
| Men, n(%) | 890 (31.3) | 1448 (43.3) | 1307 (54.9) | 1502 (64.9) | <0.001 |
| BMI kg/m2, mean (SD) | 27.60 (6.21) | 28.00 (5.78) | 27.80 (5.42) | 27.80 (5.67) | <0.001 |
| Smoking, n(%) | 1371 (49.3) | 1497 (45.6) | 970 (41.5) | 906 (40.0) | <0.001 |
| Alcohol >6 units, n(%) | 1467 (54.5) | 1773 (56.1) | 1391 (62.1) | 1425 (64.7) | <0.001 |
| SBP mmHg, mean (SD) | 166.23 (29.39) | 163.81 (28.34) | 163.36 (27.28) | 162.17 (27.47) | 0.002 |
| DBP mmHGg, mean (SD) | 96.13 (15.42) | 96.44 (14.38) | 98.03 (22.46) | 97.96 (13.87) | <0.001 |
| TC mmol/l, mean (SD) | 6.16 (1.88) | 5.90 (1.26) | 5.83 (1.16) | 5.78 (1.45) | <0.001 |
| eGFR<60, n(%) | 737 (26.2) | 796 (23.9) | 524 (22.3) | 459 (20.0) | <0.001 |
| ALP U/L, median(IQR) | 100 (71-165) | 133 (81-190) | 142 (86-193) | 141 (86-189) | <0.001 |
| ALT U/L, median(IQR) | 21 (15-30) | 23 (17-33) | 25 (18-36) | 27 (19-40) | <0.001 |
| AST U/L, median(IQR) | 21 (17-26) | 22 (18-28) | 24 (20-30) | 25 (20-32) | <0.001 |
| GGT U/L, median(IQR) | 24 (15-41) | 26 (17-42) | 28 (18-47) | 31 (19-53) | <0.001 |
| Albumin g/L, mean (SD) | 42.98 (3.76) | 43.78 (3.60) | 44.22 (3.66) | 44.53 (3.71) | <0.001 |

SD=standard deviation, BMI=body mass index, SBP=systolic blood pressure, DBP=diastolic blood pressure, TC=total cholesterol, eGFR=estimated glomerular filtration rate, Bili=bilirubin.

Table S2: Liver biochemistry based group average effect on longitudinal changes in blood pressure

| Variables | SBP | DBP |
| --- | --- | --- |
|  | GEE β, 95% CI | GEE β, 95% CI |
| **Log AST** | N=20053, G=3868 | N=20053, G=3868 |
|  | -1.59, -3.53; 0.32 | 0.60, -0.54; 1.76 |
| **Log ALT** | N=19367, G=3733 | N=19366, G=3733 |
|  | -1.94, -3.46; -0.42* | 0.74, -0.30; 1.78 |
| **Log GGT** | N=18083, G=3497 | N=18082, G=3497 |
|  | 2.92, 1.85; 3.98^†^ | 2.45, 1.73; 3.16^†^ |
| **Log ALP** | N=20328, G=3920 | N=20327, G=3920 |
|  | 8.96, 7.48; 10.45^†^ | 4.03, 3.06; 5.01^†^ |
| **Log Bilirubin** | N=19583, G=3779 | N=19582, G=3779 |
|  | -2.03, -3.78; -0.29* | 0.15, -0.99; 1.29 |

*p<0.05, ^†^p<0.001, ALT=alanine aminotransferase, AST=aspartate transaminase, ALP=alkaline phosphatase, GGT=gamma-glutamyltransferase, CI=confidence interval, REF=reference group, N=total number of observations, G=total number of individuals/groups and GEE β=generalised estimating equations β coefficient . All models are adjusted for age, gender, epochs, BMI=body mass index, tobacco smoking, alcohol use and eGFR CKD status.

Table S3: Cox-Proportional hazard model of liver enzymes and mortality events (adjusted for other liver enzymes)

| **Variables** | **All-cause**  **mortality** | | **CVD**  **mortality** | | **IHD**  **mortality** | | **Stroke**  **mortality** | | **Non-CVD mortality** | |
| --- | --- | --- | --- | --- | --- | --- | --- | --- | --- | --- |
|  | HR | 95% CI | HR | 95% CI | HR | 95% CI | HR | 95% CI | HR | 95% CI |
| **ALT** | **N=2203/8223** | | **N=1251/8223** | | **N=683/8223** | | **N=307/8277** | | **N=952/8233** | |
| Quartile 1 | 1 |  | 1 |  | 1 |  | 1 |  | 1 |  |
| Quartile 2 | 0.78* | 0.70-0.87 | 0.79* | 0.68-0.91 | 0.84 | 0.68-1.03 | 0.75 | 0.56-1.00 | 0.77* | 0.65-0.91 |
| Quartile 3 | 0.80* | 0.71-0.90 | 0.74* | 0.62-0.88 | 0.72* | 0.57-0.91 | 0.75 | 0.53-1.05 | 0.89 | 0.74-1.08 |
| Quartile 4 | 0.84* | 0.74-0.97 | 0.83* | 0.70-0.98 | 0.77* | 0.61-0.97 | 0.81 | 0.57-1.15 | 0.86 | 0.70-1.05 |
| **AST** | **N=1737/8124** | | **N=929/8124** | | **N=508/8124** | | **N=209/8124** | | **N=808/8124** | |
| Quartile 1 | 1 |  | 1 |  | 1 |  | 1 |  | 1 |  |
| Quartile 2 | 0.87* | 0.76-0.99 | 0.89 | 0.74-1.07 | 1.05 | 0.83-1.34 | 0.75 | 0.50-1.11 | 0.84 | 0.70-1.02 |
| Quartile 3 | 0.77* | 0.67-0.87 | 0.83* | 0.69-0.99 | 0.78 | 0.61-1.01 | 0.83 | 0.57-1.22 | 0.70* | 0.57-0.85 |
| Quartile 4 | 0.94 | 0.82-1.08 | 0.99 | 0.82-1.20 | 1 | 0.77-1.30 | 1.03 | 0.69-1.53 | 0.89 | 0.72-1.09 |
| **GGT** | **N=1737/8124** | | **N=929/8124** | | **N=508/8124** | | **N=209/8124** | | **N=808/8124** | |
| Quartile 1 | 1 |  | 1 |  | 1 |  | 1 |  | 1 |  |
| Quartile 2 | 0.97 | 0.85-1.11 | 0.92 | 0.76-1.10 | 0.87 | 0.68-1.12 | 0.96 | 0.66-1.44 | 1.04 | 0.86-1.27 |
| Quartile 3 | 1.02 | 0.88-1.18 | 0.97 | 0.80-1.19 | 0.85 | 0.64-1.11 | 1.22 | 0.81-1.86 | 1.08 | 0.87-1.34 |
| Quartile 4 | 1.18* | 1.02-1.38 | 1.10 | 0.90-1.36 | 0.98 | 0.74-1.29 | 1.34 | 0.87-2.07 | 1.29* | 1.03-1.62 |
| **ALP** | **N=1737/8124** | | **N=929/8124** | | **N=508/8124** | | **N=209/8124** | | **N=808/8124** | |
| Quartile 1 | 1 |  | 1 |  | 1 |  | 1 |  | 1 |  |
| Quartile 2 | 1.05 | 0.92-1.20 | 1.06 | 0.88-1.26 | 1.06 | 0.83-1.36 | 0.91 | 0.63-1.31 | 1.04 | 0.85-1.27 |
| Quartile 3 | 1.25* | 1.07-1.46 | 1.20 | 0.97-1.49 | 1.38* | 1.04-1.84 | 0.78 | 0.49-1.26 | 1.30* | 1.04-1.63 |
| Quartile 4 | 1.61* | 1.39-1.87 | 1.68* | 1.37-2.05 | 1.90* | 1.45-2.49 | 1.35 | 0.89-2.07 | 1.54* | 1.23-1.92 |
| **Bilirubin** | **N=1737/8124** | | **N=929/8124** | | **N=508/8124** | | **N=209/8124** | | **N=808/8124** | |
| Quartile 1 | 1 |  | 1 |  | 1 |  | 1 |  | 1 |  |
| Quartile 2 | 0.89 | 0.79-1.00 | 0.90 | 0.77-1.06 | 0.92 | 0.74-1.15 | 1.11 | 0.79-1.55 | 0.88 | 0.73-1.04 |
| Quartile 3 | 0.81* | 0.70-0.94 | 0.77* | 0.63-0.94 | 0.82 | 0.63-1.08 | 0.77 | 0.49-1.21 | 0.85 | 0.69-1.04 |
| Quartile 4 | 0.79* | 0.68-0.92 | 0.80* | 0.65-0.99 | 0.87 | 0.66-1.14 | 0.95 | 0.62-1.47 | 0.77* | 0.61-0.96 |

*p<0.05, ALT=alanine aminotransferase, AST=aspartate transaminase, ALP=alkaline phosphatase, GGT=gamma-glutamyltransferase (GGT)SBP=systolic blood pressure, DBP=diastolic blood pressure, CVD=Cardiovascular disease and IHD=ischemic heart disease, Non-CVD=non-cardiovascular disease, HR=hazard Ratio, CI=confidence interval. All models are adjusted for age, gender, BMI, tobacco smoking, alcohol use, year of first visit (epoch), baseline SBP and DBP, eGFR, and final achieved SBP, serum cholesterol, diabetes status and the remaining concomitant liver tests (AST was not included with ALT).

Table S4: Liver markers and all-cause mortality in sub-groups.

| Variables | Age≤55 | Age>55 | BMI≤25 | BMI>25 | Alcohol Users | Alcohol non-users |
| --- | --- | --- | --- | --- | --- | --- |
|  | HR , 95% CI | HR, 95% CI | HR, 95% CI | HR, 95% CI | HR, 95% CI | HR, 95% CI |
| **ALT** | N=671/4222 | N=902/2862 | N=471/1834 | N=1068/5133 | N=579/2987 | N=994/4097 |
| **Q1** | REF | REF | REF | REF | REF | REF |
| **Q2** | 0.89, 0.70-1.11 | 0.69, 0.58-0.81^†^ | 0.75, 0.60-0.94* | 0.71, 0.60-0.85^†^ | 0.67, 0.54-0.82^†^ | 0.81, 0.68-0.97* |
| **Q3** | 0.88, 0.69-1.12 | 0.66, 0.54-0.81^†^ | 0.60, 0.42-0.77^†^ | 0.78, 0.65-0.94* | 0.75, 0.58-0.95* | 0.75, 0.61-0.92* |
| **Q4** | 0.78, 0.60-1.01 | 0.64, 0.51-0.80^†^ | 0.65, 0.46-0.91* | 0.68, 0.56-0.83^†^ | 0.70, 0.52-0.94* | 0.69, 0.55-0.85^†^ |
| **AST** | N=696/4892 | N=1041/3232 | N=530/2111 | N=1174/5879 | N=683/3498 | N=1054/4626 |
| **Q1** | 1.17, 0.94-1.46 | 1.17, 0.99-1.37 | 1.33, 1.05-1.69* | 1.09, 0.93-1.28 | 1.33, 1.09-1.63* | 1.01, 0.85-1.20 |
| **Q2** | REF | REF | REF | REF | REF | REF |
| **Q3** | 1.00, 0.80-1.26 | 0.82, 0.69-0.98* | 1.04, 0.81-1.34 | 0.81, 0.69-0.96* | 0.95, 0.76-1.17 | 0.84, 0.70-1.00* |
| **Q4** | 1.14, 0.91-1.42 | 1.05, 0.87-1.27 | 1.22, 0.93-1.60 | 1.000, 0.84-1.18 | 1.08, 0.85-1.38 | 1.04, 0.87-1.24 |
| **GGT** | N=605/3993 | N=862/2733 | N=447/1767 | N=988/4849 | N=558/2906 | N=909/3820 |
| **Q1** | REF | REF | REF | REF | REF | REF |
| **Q2** | 0.93, 0.74-1.18 | 0.93, 0.78-1.12 | 1.08, 0.84-1.37 | 0.89, 0.74-1.06 | 0.93, 0.75-1.15 | 0.96, 0.79-1.17 |
| **Q3** | 1.13, 0.88-1.44 | 0.91, 0.74-1.13 | 1.27, 0.96-1.67 | 0.92, 0.76-1.12 | 0.87, 0.68-1.13 | 1.09, 0.89-1.33 |
| **Q4** | 1.10, 0.85-1.43 | 1.10, 0.86-1.39 | 1.54, 1.11-2.13* | 1.02, 0.83-1.27 | 0.82, 0.60-1.13 | 1.30, 1.05-1.61* |
| **ALP** | N=618/3994 | N=791/2539 | N=412/1706 | N=968/4720 | N=482/2626 | N=927/3907 |
| **Q1** | REF | REF | REF | REF | REF | REF |
| **Q2** | 1.09, 0.89-1.33 | 1.04, 0.86-1.26 | 0.93, 0.73-1.20 | 1.15, 0.97-1.35 | 1.15, 0.90-1.48 | 1.04, 0.89-1.23 |
| **Q3** | 1.24, 0.98-1.57 | 1.18, 0.94-1.48 | 0.99, 0.74-1.34 | 1.34, 1.10-1.64* | 1.39, 1.05-1.84* | 1.17, 0.95-1.43 |
| **Q4** | 1.59, 1.22-2.06* | 1.24, 0.97-1.58 | 0.99, 0.71-1.38 | 1.55, 1.25-1.95^†^ | 1.41, 1.03-1.95* | 1.40, 1.09-1.69* |
| **Bilirubin** | N=679/4278 | N=907/2879 | N=473/1839 | N=1079/5201 | N=580/3003 | N=1006/4154 |
| **Q1** | REF | REF | REF | REF | REF | REF |
| **Q2** | 0.83, 0.68-1.01 | 0.95, 0.80-1.12 | 0.92, 0.73-1.16 | 0.87, 0.74-1.01 | 0.75, 0.61-0.93^†^ | 0.99, 0.84-1.16 |
| **Q3** | 0.76, 0.61-0.96* | 0.89, 0.73-1.08 | 0.85, 0.65-1.12 | 0.80, 0.67-0.96* | 0.65, 0.50-0.84^†^ | 0.94, 0.78-1.13 |
| **Q4** | 0.79, 0.63-0.99* | 0.79, 0.64-0.99* | 0.72, 0.54-0.96* | 0.79, 0.65-0.96* | 0.63, 0.48-0.83^†^ | 0.90, 0.75-1.10 |

*p<0.05, ^†^p<0.001, ALT=alanine aminotransferase, AST=aspartate transaminase, ALP=alkaline phosphatase, GGT=gamma-glutamyltransferase (GGT), CI=confidence interval, REF=reference group, and HR=hazard ratio.

Table S5. Incremental utility of liver function tests biomarkers on cardiovascular mortality in hypertensive patients

| Variables | AUC  [95% CI] | AUC Diff | NRI(Categorical)  [95% CI] | P | NRI(Continuous)  [95% CI] | P | IDI  [95% CI] | P |
| --- | --- | --- | --- | --- | --- | --- | --- | --- |
| **35 Year Follow-up** | | | | | | | | |
| Base | 0.824  [0.809 - 0.839] |  |  |  |  |  |  |  |
| Base + Bilirubin | 0.827  [0.812 - 0.842] | 0.003 | 0.0114  [-0.0036-0.0265] | 0.136 | **0.1654**  **[0.0822-0.2486]** | **0.0001** | **0.0061**  **[0.0025-0.0098]** | **0.0011** |
| Base + ALP + Bilirubin | 0.831  [0.816 - 0.846] | 0.007 | **0.0288**  **[0.0079-0.0496]** | **0.00687** | **0.2986**  **[0.2154-0.3817]** | **<0.0001** | **0.0153**  **[0.0094-0.0211]** | **<0.0001** |
| Base + LFT | 0.83  [0.815 - 0.845] | 0.006 | 0.0205  [-0.0012-0.0423] | 0.064 | **0.2907**  **[0.2075-0.3738]** | **<0.0001** | **0.0162**  **[0.0097-0.0227]** | **<0.0001** |
| **20 Year Follow-up** | | | | | | | | |
| Base | 0.833  [0.817 - 0.85] |  |  |  |  |  |  |  |
| Base + Bilirubin | 0.837  [0.821 - 0.853] | 0.004 | 0.0148  [-0.0107-0.0404] | 0.255 | **0.1759**  **[0.0849-0.2669]** | **0.00015** | **0.0049**  **[0.0013-0.0086]** | **0.0085** |
| Base + ALP + Bilirubin | 0.84  [0.824 - 0.856] | 0.007 | 0.0204  [-0.0092-0.0501] | 0.17718 | **0.3042**  **[0.2133-0.3952]** | **<0.0001** | **0.0111**  **[0.0055-0.0167]** | **0.00011** |
| Base + LFT | 0.839  [0.823 - 0.855] | 0.006 | 0.0056  [-0.0252-0.0365] | 0.720 | **0.2816**  **[0.1906-0.3726]** | **<0.0001** | **0.013**  **[0.0067-0.0194]** | **0.00005** |
| **10 Year Follow-up** | | | | | | | | |
| Base | 0.827  [0.804 - 0.85] |  |  |  |  |  |  |  |
| Base + Bilirubin | 0.828  [0.804 - 0.851] | 0.001 | -0.0141  [-0.0558-0.0276] | 0.507 | 0.0497  [-0.0758-0.1752] | 0.437 | **0.0048**  **[0.001-0.0086]** | **0.0126** |
| Base + ALP + Bilirubin | 0.833  [0.811 - 0.856] | 0.006 | 0.0102  [-0.035-0.0554] | 0.65965 | **0.2189**  **[0.0934-0.3444]** | **0.00063** | **0.0088**  **[0.0031-0.0145]** | **0.00242** |
| Base + LFT | 0.835  [0.813 - 0.857] | 0.008 | -0.0043  [-0.0568-0.0483] | 0.873 | **0.3533**  **[0.2278-0.4788]** | **<0.0001** | **0.0138**  **[0.0065-0.0211]** | **0.0002** |

Baseline model includes age, sex, systolic blood pressure, diastolic blood pressure, smoking status, alcohol use, body mass index, Cholesterol, epochs and chronic kidney disease status. AUC=Area under the curve, NRI=Net reclassification index, P=p value, IDI=Integrated descrimination index, ALP=alkaline phosphatase, LFT=Liver function tests, and CI=Confidence interval.

Table S6: Full risk discrimination and reclassification analysis

| Risk model- FU=35 years | AUC  [95% CI] | AUC Difference | NRI(Categorical)  [95% CI] | P | NRI(Continuous)  [95% CI] | P | IDI  [95% CI] | P |
| --- | --- | --- | --- | --- | --- | --- | --- | --- |
| base | 0.824  [0.809 - 0.839] |  |  |  |  |  |  |  |
| base+Albumin | 0.824  [0.809 - 0.839] | 0 | 0.003  [-0.01-0.016] | 0.646 | -0.06  [-0.143-0.023] | 0.157 | 2e-04  [-0.002-0.003] | 0.849 |
| base+ALT | 0.824  [0.808 - 0.839] | 0 | 0.004  [-0.007-0.015] | 0.452 | 0.083  [-7e-04-0.166] | 0.051 | 0.001  [-3e-04-0.003] | 0.109 |
| base+AST | 0.824  [0.809 - 0.839] | 0 | 0.004  [-0.005-0.013] | 0.343 | 0.047  [-0.037-0.130] | 0.272 | 7e-04  [-5e-04-0.002] | 0.277 |
| base+ALP | 0.827  [0.811 - 0.842] | 0.003 | -4e-04  [-0.017-0.016] | 0.962 | 0.074  [-0.009-0.157] | 0.081 | **0.008**  **[0.004-0.012]** | **<0.001** |
| base+GGT | 0.824  [0.808 - 0.839] | 0 | -0.004  [-0.013-0.006] | 0.457 | 0.008  [-0.075-0.092] | 0.844 | -1e-04  [-9e-04-6e-04] | 0.755 |
| base+Bilirubin | 0.827  [0.812 - 0.842] | 0.003 | 0.011  [-0.004-0.027] | 0.136 | **0.165**  **[0.082-0.249]** | **<0.001** | **0.006**  **[0.003-0.010]** | **0.001** |
| base+GGT+Bilirubin | 0.827  [0.812 - 0.842] | 0.003 | 0.002  [-0.014-0.019] | 0.766 | **0.143**  **[0.059-0.226]** | **<0.001** | **0.006**  **[0.002-0.010]** | **0.003** |
| base+Albumin+Bilirubin | 0.827  [0.812 - 0.842] | 0.003 | 0.010  [-0.005-0.027] | 0.192 | **0.119**  **[0.036-0.202]** | **0.0051** | **0.006**  **[0.002-0.01]** | **0.004** |
| **base+ALP+Bilirubin** | 0.831  [0.816 - 0.846] | 0.007 | **0.029**  **[0.008-0.050]** | **0.007** | **0.299**  **[0.215-0.382]** | **<0.001** | **0.015**  **[0.009-0.021]** | **<0.001** |
| base+ALT+Bilirubin | 0.827  [0.812 - 0.842] | 0.003 | 0.010  [-0.007-0.026] | 0.249 | **0.172**  **[0.089-0.255]** | **<0.001** | **0.007**  **[0.003-0.011]** | **<0.001** |
| base+AST+Bilirubin | 0.827  [0.812 - 0.842] | 0.003 | 0.011  [-0.005-0.026] | 0.170 | **0.170**  **[0.086-0.253]** | **0<0.001** | **0.006**  **[0.003-0.010]** | **<0.001** |
| base+GGT+Albumin | 0.823  [0.808 - 0.839] | -0.001 | 0.007  [-0.007-0.020] | 0.324 | -0.015  [-0.098-0.068] | 0.723 | 1e-04  [-0.003-0.003] | 0.927 |
| base+ALP+Albumin | 0.827  [0.812 - 0.842] | 0.003 | 6e-04  [-0.019-0.020] | 0.949 | **0.159**  **[0.075-0.242]** | **<0.001** | **0.008**  **[0.004-0.013]** | **<0.001** |
| base+ALT+Albumin | 0.824  [0.809 - 0.839] | 0 | 0.011  [-0.003-0.024] | 0.126 | 0.034  [-0.050-0.117] | 0.430 | 0.001  [-0.001-0.004] | 0.287 |
| base+AST+Albumin | 0.824  [0.808 - 0.839] | 0 | 0.003  [-0.011-0.017] | 0.667 | 0.027  [-0.057-0.110] | 0.531 | 8e-04  [-0.002-0.003] | 0.530 |
| base+GGT+ALT | 0.823  [0.808 - 0.839] | -0.001 | -8e-04  [-0.013-0.011] | 0.887 | **0.095**  **[0.012-0.178]** | **0.025** | 0.002  [-7e-04-0.004] | 0.189 |
| base+ALP+ALT | 0.827  [0.811 - 0.842] | 0.003 | 0.015  [-0.002-0.032] | 0.086 | 0.107  [0.024-0.191] | 0.011 | **0.009**  **[0.005-0.014]** | **<0.001** |
| 35 base+ALP+GGT | 0.827  [0.811 - 0.842] | 0.003 | -4e-04  [-0.017-0.016] | 0.963 | 0.071  [-0.012-0.154] | 0.095 | **0.008**  **[0.004-0.012]** | **<0.001** |
| 35 base+AST+GGT | 0.823  [0.808 - 0.839] | -0.001 | 0.002  [-0.007-0.011] | 0.614 | 0.014  [-0.069-0.098] | 0.735 | 6e-04  [-0.001-0.002] | 0.467 |
| 35 base+AST+ALP | 0.826  [0.811 - 0.842] | 0.002 | 0.011  [-0.007-0.028] | 0.236 | **0.111**  **[0.028-0.195]** | **0.009** | **0.009**  **[0.004-0.013]** | **<0.001** |
| **35 base+LFT** | 0.83  [0.815 - 0.845] | 0.006 | 0.021  [-0.001-0.042] | 0.064 | **0.291**  **[0.208-0.374]** | **<0.001** | **0.016**  **[0.010-0.023]** | **<0.001** |
|  |  |  |  |  |  |  |  |  |
| Risk model- FU=20 years | AUC  [95% CI]for the model 2 |  | NRI(Categorical)  [95% CI] | P | NRI(Continuous)  [95% CI] | P | IDI  [95% CI] | P |
| base | 0.833  [0.817 - 0.85] |  |  |  |  |  |  |  |
| base+Albumin | 0.833  [0.817 - 0.849] | 0 | -0.002  [-0.021-0.017] | 0.841 | -0.040  [-0.131-0.051] | 0.386 | 9e-04  [-0.002-0.003] | 0.479 |
| base+ALT | 0.833  [0.816 - 0.849] | 0 | -0.008  [-0.022-0.007] | 0.303 | 0.041  [-0.049-0.132] | 0.374 | **0.001**  **[1e-04-0.003]** | **0.028** |
| base+AST | 0.833  [0.817 - 0.849] | 0 | -0.011  [-0.023-0.001] | 0.075 | 0.004  [-0.087-0.095] | 0.934 | 5e-04  [-1e-04-0.001] | 0.124 |
| base+ALP | 0.836  [0.82 - 0.852] | 0.003 | -0.002  [-0.025-0.020] | 0.836 | **0.102**  **[0.011-0.193]** | **0.028** | **0.005**  **[0.002-0.008]** | **0.005** |
| base+GGT | 0.833  [0.817 - 0.85] | 0 | 0.003  [-0.013-0.019] | 0.676 | 0.066  [-0.025-0.157] | 0.156 | -1e-04  [-0.0011-8e-04] | 0.767 |
| base+Bilirubin | 0.837  [0.821 - 0.853] | 0.004 | 0.015  [-0.010-0.040] | 0.255 | **0.176**  **[0.085-0.267]** | **<0.001** | **0.005**  **[0.001-0.009]** | **0.009** |
| base+GGT+Bilirubin | 0.837  [0.821 - 0.853] | 0.004 | 0.015  [-0.010-0.042] | 0.249 | **0.149**  **[0.058-0.240]** | **0.001** | **0.005**  **[9e-04-0.009]** | **0.016** |
| base+Albumin+Bilirubin | 0.837  [0.82 - 0.853] | 0.004 | 0.008  [-0.017-0.034] | 0.526 | **0.110**  **[0.019-0.200]** | **0.018** | **0.005**  **[0.001-0.010]** | **0.011** |
| **base+ALP+Bilirubin** | 0.84  [0.824 - 0.856] | 0.007 | 0.020  [-0.009-0.050] | 0.177 | **0.304**  **[0.213-0.395]** | **<0.001** | **0.011**  **[0.006-0.017]** | **<0.001** |
| base+ALT+Bilirubin | 0.837  [0.821 - 0.853] | 0.004 | 0.011  [-0.014-0.037] | 0.388 | **0.140**  **[0.049-0.231]** | **0.003** | **0.006**  **[0.002-0.010]** | **0.002** |
| base+AST+Bilirubin | 0.837  [0.821 - 0.853] | 0.004 | 0.013  [-0.012-0.038] | 0.306 | **0.156**  **[0.065-0.247]** | **<0.001** | **0.005**  **[0.001-0.009]** | **0.007** |
| base+GGT+Albumin | 0.833  [0.817 - 0.849] | 0 | 0.011  [-0.010-0.032] | 0.314 | 0.018  [-0.073-0.109] | 0.702 | 9e-04  [-0.002-0.004] | 0.550 |
| base+ALP+Albumin | 0.836  [0.82 - 0.852] | 0.003 | 0.005  [-0.021-0.031] | 0.707 | **0.198**  **[0.107-0.289]** | **<0.001** | **0.006**  **[0.002-0.010]** | **0.005** |
| base+ALT+Albumin | 0.833  [0.816 - 0.849] | 0 | 0.003  [-0.018-0.024] | 0.771 | 0.018  [-0.074-0.109] | 0.706 | 0.002  [-6e-04-0.005] | 0.120 |
| base+AST+Albumin | 0.833  [0.817 - 0.849] | 0 | 0.002  [-0.018-0.022] | 0.847 | -0.018  [-0.109-0.073] | 0.698 | 0.001  [-0.001-0.004] | 0.297 |
| base+GGT+ALT | 0.833  [0.816 - 0.849] | 0 | -0.008  [-0.026-0.010] | 0.393 | **0.097**  **[0.006-0.188]** | **0.036** | 0.002  [-2e-04-0.004] | 0.078 |
| base+ALP+ALT | 0.835  [0.819 - 0.851] | 0.002 | -0.014  [-0.037-0.009] | 0.221 | **0.129**  **[0.038-0.22]** | **0.005** | **0.007**  **[0.003-0.010]** | **<0.001** |
| base+ALP+GGT | 0.836  [0.82 - 0.852] | 0.003 | 0.002  [-0.021-0.026] | 0.848 | **0.107**  **[0.016-0.198]** | **0.020** | **0.005**  **[0.001-0.008]** | **0.007** |
| base+AST+GGT | 0.833  [0.817 - 0.849] | 0 | -0.010  [-0.027-0.008] | 0.266 | 0.033  [-0.058-0.124] | 0.476 | 6e-04  [-8e-04-0.002] | 0.407 |
| base+AST+ALP | 0.835  [0.819 - 0.852] | 0.002 | -0.009  [-0.031-0.014] | 0.435 | **0.157**  **[0.066-0.248]** | **<0.001** | **0.006**  **[0.002-0.009]** | **0.001** |
| **base+LFT** | 0.839  [0.823 - 0.855] | 0.006 | 0.006  [-0.025-0.037] | 0.720 | **0.282**  **[0.191-0.373]** | **<0.001** | **0.013**  **[0.007-0.019]** | **<0.001** |
|  |  |  |  |  |  |  |  |  |
| Risk model- FU=10 years | AUC  [95% CI]for the model 2 |  | NRI(Categorical)  [95% CI] | P | NRI(Continuous)  [95% CI] | P | IDI  [95% CI] | P |
| base | 0.827  [0.804 - 0.85] |  |  |  |  |  |  |  |
| base+Albumin | 0.828  [0.805 - 0.851] | 0.001 | -0.018  [-0.055-0.019] | 0.348 | 0.129  [0.003-0.254] | 0.044 | 0.003  [-8e-04-0.007] | 0.117 |
| base+ALT | 0.827  [0.803 - 0.85] | 0 | 0.012  [-0.010-0.034] | 0.273 | 0.060  [-0.065-0.186] | 0.347 | **0.002**  **[2e-04-0.003]** | **0.024** |
| base+AST | 0.827  [0.803 - 0.85] | 0 | 0.019  [-0.013-0.051] | 0.250 | 0.085  [-0.041-0.210] | 0.186 | **0.003**  **[8e-04-0.005]** | **0.007** |
| base+ALP | 0.832  [0.809 - 0.855] | 0.005 | 0.009  [-0.028-0.045] | 0.645 | **0.184**  **[0.058-0.309]** | **0.004** | 0.004  [-2e-04-0.008] | 0.066 |
| base+GGT | 0.828  [0.804 - 0.851] | 0.001 | 0.005  [-0.014-0.024] | 0.596 | 0.085  [-0.040-0.211] | 0.184 | 1e-04  [-0.001-0.002] | 0.890 |
| base+Bilirubin | 0.828  [0.804 - 0.851] | 0.001 | -0.014  [-0.056-0.028] | 0.508 | 0.050  [-0.076-0.175] | 0.437 | **0.005**  **[0.001-0.009]** | **0.013** |
| base+GGT+Bilirubin | 0.828  [0.805 - 0.851] | 0.001 | -0.009  [-0.051-0.033] | 0.668 | 0.087  [-0.038-0.213] | 0.172 | **0.005**  **[8e-04-0.010]** | **0.021** |
| base+Albumin+Bilirubin | 0.829  [0.805 - 0.852] | 0.002 | -0.007  [-0.051-0.037] | 0.745 | **0.126**  **[5e-04-0.252]** | **0.049** | **0.007**  **[0.002-0.013]** | **0.006** |
| base+ALP+Bilirubin | 0.833  [0.811 - 0.856] | 0.006 | 0.0102  [-0.035-0.055] | 0.660 | **0.219**  **[0.093-0.344]** | **<0.001** | **0.009**  **[0.003-0.015]** | **0.002** |
| base+ALT+Bilirubin | 0.828  [0.804 - 0.851] | 0.001 | -0.023  [-0.061-0.014] | 0.219 | 0.027  [-0.099-0.152] | 0.674 | **0.006**  **[0.002-0.010]** | **0.003** |
| base+AST+Bilirubin | 0.827  [0.804 - 0.851] | 0 | 0.003  [-0.039-0.044] | 0.899 | 0.121  [-0.005-0.246] | 0.059 | **0.007**  **[0.003-0.011]** | **<0.001** |
| base+GGT+Albumin | 0.828  [0.806 - 0.851] | 0.001 | 0.009  [-0.032-0.050] | 0.663 | 0.162  [0.037-0.287] | 0.011 | 0.004  [-7e-04-0.008] | 0.099 |
| **base+ALP+Albumin** | 0.834  [0.812 - 0.857] | 0.007 | -0.007  [-0.048-0.034] | 0.744 | **0.309**  **[0.184-0.435]** | **<0.001** | **0.007**  **[0.002-0.013]** | **0.013** |
| base+ALT+Albumin | 0.828  [0.805 - 0.851] | 0.001 | -0.018  [-0.0566-0.021] | 0.368 | 0.115  [-0.010-0.240] | 0.073 | **0.004**  **[3e-04-0.009]** | **0.037** |
| base+AST+Albumin | 0.828  [0.805 - 0.851] | 0.001 | -0.007  [-0.049-0.036] | 0.754 | 0.108  [-0.017-0.234] | 0.091 | **0.006**  **[0.001-0.010]** | **0.010** |
| base+GGT+ALT | 0.828  [0.804 - 0.851] | 0.001 | -0.007  [-0.036-0.022] | 0.648 | **0.154**  **[0.029-0.280]** | **0.017** | **0.003**  **[2e-04-0.005]** | **0.034** |
| base+ALP+ALT | 0.832  [0.809 - 0.855] | 0.005 | 0.008  [-0.032-0.048] | 0.691 | **0.207**  **[0.082-0.333]** | **0.001** | **0.005**  **[0.001-0.010]** | **0.012** |
| base+ALP+GGT | 0.832  [0.809 - 0.855] | 0.005 | -7e-04  [-0.037-0.036] | 0.969 | **0.211**  **[0.085-0.336]** | **0.001** | 0.004  [-4e-04-0.008] | 0.080 |
| base+AST+GGT | 0.827  [0.804 - 0.851] | 0 | 0.001  [-0.037-0.040] | 0.942 | 0.074  [-0.052-0.199] | 0.250 | **0.004**  **[9e-04-0.007]** | **0.010** |
| base+AST+ALP | 0.832  [0.809 - 0.855] | 0.005 | 0.007  [-0.037-0.051] | 0.762 | **0.241**  **[0.116-0.367]** | **<0.001** | **0.007**  **[0.002-0.011]** | **0.003** |
| **base+LFT** | 0.835  [0.813 - 0.857] | 0.008 | -0.004  [-0.057-0.048] | 0.873 | **0.353**  **[0.228-0.479]** | **<0.001** | **0.014**  **[0.007-0.021]** | **0.0002** |

Baseline model includes age, sex, systolic blood pressure, diastolic blood pressure, smoking status, alcohol use, body mass index, Cholesterol, epochs and chronic kidney disease status. ALT=alanine aminotransferase, AST=aspartate transaminase, ALP=alkaline phosphatase, GGT=gamma-glutamyltransferase (GGT), AUC=area under the ROC curve, FU=follow-up in years, CI=confidence interval, P=p value, NRI=net reclassification index, IDI=integrated discrimination index.

Supplementary Figure S1: Study flow chart

Excluded (n=408): ALT>100 U/L

N=12000

Glasgow Blood Pressure Clinic (GPBC) Database: N=16011

Excluded (n=4011): Missing data on any of the five liver enzymes

ALP*: N=11426

N=10385

Q1: 2751

Q2: 2957

Q3: 2828

Q4: 1849

Excluded (n=1041): ALP>240 U/L.

ALT*: N=10125

N=9717

Q1: 2168

Q2: 3034

Q3: 2184

Q4: 2331

AST*: N=11257

N=11059

Q1: 2794

Q2: 2582

Q3: 2945

Q4: 2738

Excluded (n=198): AST>100 U/l.

GGT*: N=10551

N=9789

Q1: 2385

Q2: 3040

Q3: 2384

Q4: 1980

Excluded (n=762): GGT>100 U/L.

Bilirubin*: N=11035

N=10890

Q1: 2845

Q2: 3348

Q3: 2381

Q4: 2316

Excluded (n=145): Bili>30 µmol/L

*Numbers included in the cubic regression spline models.

ALT=alanine aminotransferase, AST=aspartate transaminase, ALP=alkaline phosphatase, GGT=gamma-glutamyltransferase (GGT)


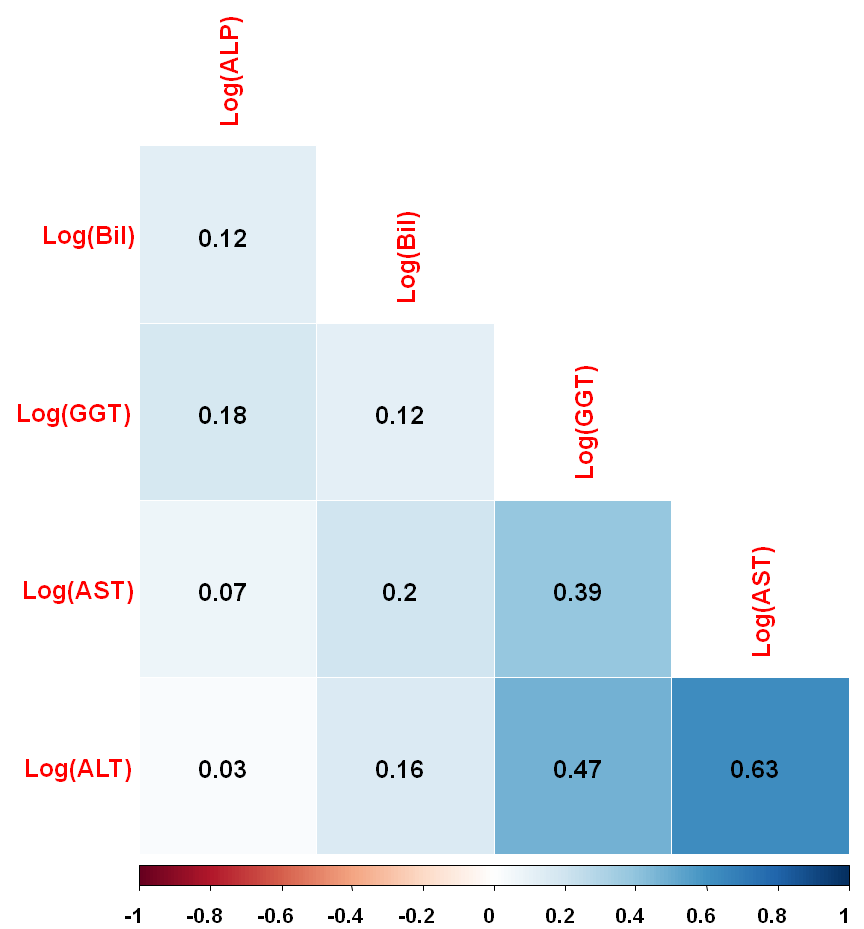
Supplementary Figure S2: Bivariate correlations between liver enzymes and bilirubin

ALT=alanine aminotransferase, AST=aspartate transaminase, ALP=alkaline phosphatase, GGT=gamma-glutamyltransferase (GGT)

Supplementary Figure S3: Liver biochemistry and longitudinal change in SBP. The rate of change in systolic blood pressure with liver parameters and their 95% confidence limits are presented. The results are stratified by potential confounding factors.


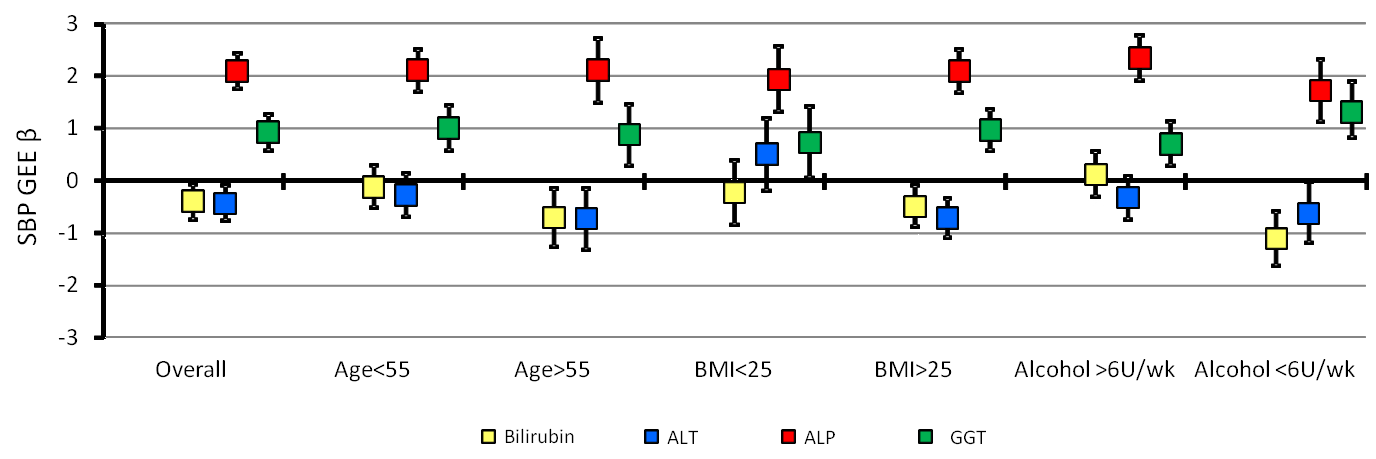


Supplementary Figure S4: Cox proportional hazard model for markers of liver dysfunction and all-cause mortality before and after imputation of missing data. It is a forest plot representing the point estimates of hazard ratios and their 95% confidence intervals.
